# Impact of extrinsic incubation temperature on natural selection during Zika virus infection of *Aedes aegypti* and *Aedes albopictus*

**DOI:** 10.1371/journal.ppat.1009433

**Published:** 2021-11-09

**Authors:** Reyes A. Murrieta, Selene M. Garcia-Luna, Deedra J. Murrieta, Gareth Halladay, Michael C. Young, Joseph R. Fauver, Alex Gendernalik, James Weger-Lucarelli, Claudia Rückert, Gregory D. Ebel

**Affiliations:** 1 Department of Microbiology, Immunology and Pathology, College of Veterinary Medicine and Biomedical Sciences, Colorado State University, Fort Collins, Colorado, United States of America; 2 Department of Entomology, Texas A&M University, College Station, Texas, United States of America; 3 Yale School of Public Health, Department of Epidemiology of Microbial Diseases, Laboratory of Epidemiology of Public Health, New Haven, Connecticut, United States of America; 4 Department of Biomedical Sciences & Pathobiology, Virginia-Maryland College of Veterinary Medicine, Virginia Tech, Blacksburg, Virginia, United States of America; 5 Department of Biochemistry and Molecular Biology, College of Agriculture, Biotechnology & Natural Resources, University of Nevada, Reno, Nevada, United States of America; NYU Langone Health, UNITED STATES

## Abstract

Arthropod-borne viruses (arboviruses) require replication across a wide range of temperatures to perpetuate. While vertebrate hosts tend to maintain temperatures of approximately 37°C—40°C, arthropods are subject to ambient temperatures which can have a daily fluctuation of > 10°C. Temperatures impact vector competence, extrinsic incubation period, and mosquito survival unimodally, with optimal conditions occurring at some intermediate temperature. In addition, the mean and range of daily temperature fluctuations influence arbovirus perpetuation and vector competence. The impact of temperature on arbovirus genetic diversity during systemic mosquito infection, however, is poorly understood. Therefore, we determined how constant extrinsic incubation temperatures of 25°C, 28°C, 32°C, and 35°C control Zika virus (ZIKV) vector competence and population dynamics within *Aedes aegypti* and *Aedes albopictus* mosquitoes. We also examined fluctuating temperatures which better mimic field conditions in the tropics. We found that vector competence varied in a unimodal manner for constant temperatures peaking between 28°C and 32°C for both *Aedes* species. Transmission peaked at 10 days post-infection for *Aedes aegypti* and 14 days for *Aedes albopictus*. Conversely, fluctuating temperature decreased vector competence. Using RNA-seq to characterize ZIKV population structure, we identified that temperature alters the selective environment in unexpected ways. During mosquito infection, constant temperatures more often elicited positive selection whereas fluctuating temperatures led to strong purifying selection in both *Aedes* species. These findings demonstrate that temperature has multiple impacts on ZIKV biology, including major effects on the selective environment within mosquitoes.

## Author summary

Arthropod-borne viruses (arboviruses) have emerged in recent decades due to complex factors that include increases in international travel and trade, the breakdown of public health infrastructure, land use changes, and many others. Climate change also has the potential to shift the geographical ranges of arthropod vectors, consequently increasing the global risk of arbovirus infection. Changing temperatures may alter the virus-host interaction, ultimately resulting in the emergence of new viruses and virus genotypes in new areas. Therefore, we sought to characterize how temperature (both constant and fluctuating) alters the ability of *Aedes aegypti* and *Aedes albopictus* to transmit Zika virus, and how it influences virus populations within mosquitoes. We found that intermediate temperatures maximize virus transmission compared to more extreme and fluctuating temperatures. Constant temperatures increased positive selection on virus genomes, while fluctuating temperatures strengthened purifying selection. Our studies provide evidence that in addition to altering vector competence, temperature significantly influences natural selection within mosquitoes.

## Introduction

Arthropod-borne viruses (arboviruses) such as Zika virus (ZIKV, Flaviviridae, *Flavivirus*) are largely RNA viruses that are transmitted by arthropod vectors among vertebrate hosts [1]. Thus, arboviruses are required to alternately replicate in hosts with drastically different body temperatures. This affects transmission dynamics, replication rates, and population structure [2–4]. While replication in vertebrates generally occurs within 2–3 degrees of 38°C [5], infection in mosquitoes may occur at a much wider range of temperatures. Mosquito vectors are distributed throughout tropical and temperate climates and the geographical range of relevant species is increasing [6]. Climate variations such as heat waves, cold snaps, or daily temperature fluctuations change the host environment within which arboviruses replicate and are transmitted. Fluctuations in the temperature of the host environment are central to arbovirus biology [7] and virus-host interaction [8–10].

The impact of temperature on vector competence (VC), i.e. the ability of a mosquito to acquire, maintain, and transmit a pathogen, is well described. In regards to ZIKV, temperature impacts VC in a unimodal manner, with extreme low (16°C) and high (38°C) temperatures associated with lower VC while median temperatures (28°C-32°C) are associated with higher VC [11,12]. For other arboviruses (West Nile virus, St. Louis Encephalitis virus, and Rift Valley Fever) extrinsic incubation temperature (EIT) has shown to influence viral replication and dissemination within vectors [13–18], which may alter the extrinsic incubation period, i.e. the number of days between acquisition of an infection and infectiousness to a new host [8,19–21]. Many studies examining the effects of EIT on VC have used single, constant EITs to represent optimal conditions for mosquito colony survival [11,12,22–25]. However, fluctuating EIT more accurately models the environmental conditions encountered by mosquitoes in the field [26–29]. Although temperature clearly exerts a strong selective pressure on RNA viruses [30,31], little is known about how it may influence the composition of arbovirus populations during mosquito infection. Thus, while EIT clearly effects arbovirus transmission and epidemiology, its impact on arbovirus evolution remains unclear.

RNA viruses like ZIKV have the capacity to evolve rapidly in response to changing environments. This is due, in part, to short generation times and error-prone replication [32,33]. As a result, arboviruses, including ZIKV, exist within hosts as large populations of mixed haplotypes, which is critical to their perpetuation in nature [34–38]. While there have been numerous studies assessing ZIKV VC and viral ecology and some efforts focusing on the use of environmental data to predict virus spread, there is limited knowledge as to how environmental factors such as EIT impact the selective environments and mutational diversity of arboviruses within mosquitoes [39]. Accordingly, we sought to determine whether ZIKV mutational diversity is altered by EIT during systemic infection of *Aedes aegypti* (*Ae*. *aegypti)* and *Aedes albopictus* (*Ae*. *albopictus*) vectors. We exposed mosquitoes to a Puerto Rican isolate of ZIKV and held them at constant EITs of 25°C, 28°C, 32°C, 35°C; and a temperature that fluctuated between 25°C to 35°C every 12 hours. We then assessed VC and measured virus mutational diversity in different tissue compartments of each mosquito using next-generation sequencing (NGS). Our results suggest that the selective environment within mosquitoes is significantly modified by EIT, and that EIT fluctuations exert unique constraints upon arbovirus sequences.

## Results

### Vector competence

**Fig 1:**
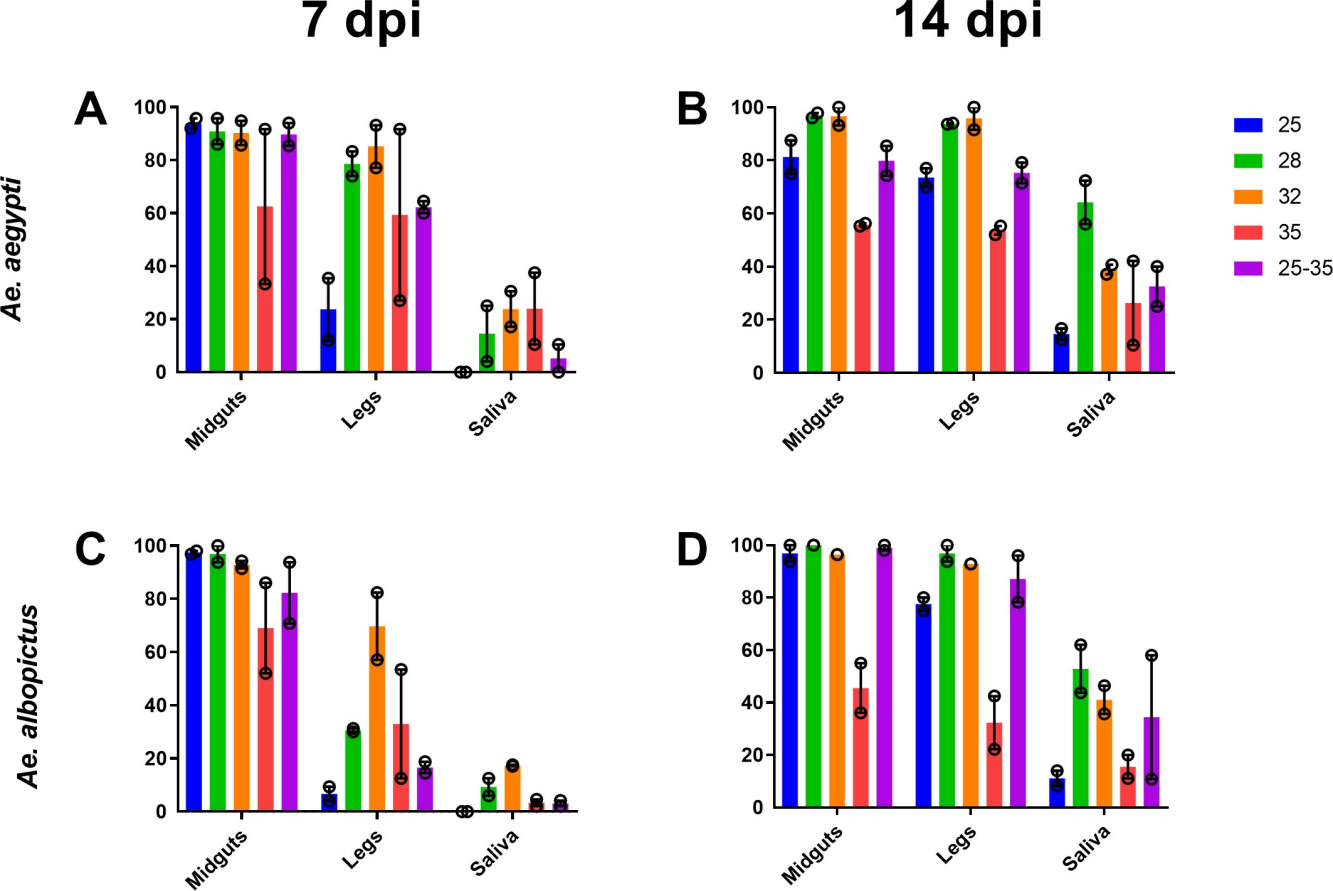
Extrinsic incubation temperature alters ZIKV transmission efficiency in *Aedes* mosquitoes. Percent of *Ae*. *aegypti* (average number per EIT group = 86, S3 Table) (A & B) and *Ae*. *albopictus* (average number per EIT group = 80, S3 Table) (C & D) with ZIKV in midgut, legs, and saliva at 7 (A & C) and 14 (B&D) days post feeding. The bar represents the mean, and the open circles represent the value of each experiment with SEM shown with error bars.

To assess how EIT affects vector competence for ZIKV, we exposed *Ae*. *aegypti* and *Ae*. *albopictus* to ZIKV (n = 72–108) and held them at 25°C, 28°C, 32°C, 35°C, and temperatures that fluctuated between 25°C-35°C. Infection rates were high in all mosquitoes except those held at 35°C (Fig 1). Data was evaluated using generalized linear models (GLM). Three models were constructed for VC (infection, dissemination, and transmission). The best fit generalized linear models (S1 Table) for infection and dissemination was the full model incorporating days post infection (dpi), species, and both the linear and quadratic effects of temperature.


$$vector\;competence∼exp\;[\beta_{1}(dpi)\times \beta_{2}(species)\times \beta_{3}(temp)+{\beta_{4}(temp)}^{2}]$$

The best fit model for transmission was a reduced version:

$$vector\;competence∼exp\;[\beta_{1}(dpi)+\beta_{2}(species)+\beta_{3}(temp)+{\beta_{4}(temp)}^{2}+\beta_{5}(dpi:temp)+\beta_{6}(species:temp)]$$

Vector competence for all three tissues was significantly impacted by all factors and associated interactions (where noted in model). In both *Aedes* species, EIT impacted vector competence in a unimodal manner with moderate EITs (28°C and 32°C) showing an increased dissemination, transmission, and infection with other factors held constant (Fig 1A–1D and S2 Table). The difference in dissemination was most notable in *Ae*. *albopictus*, which was ~30% dissemination at 28°C and ~80% at 32°C (Fig 1A). VC for the fluctuating EIT group was compared with averaged VC values for static EIT groups 28°C and 32°C, due to those EITs most closely resembling optimal mean EIT. The fluctuating EIT group did not fit with the expected unimodal distribution given the mean daily EIT in this group (30°C, S3 Table). Instead, infection in the fluctuating EIT group was significantly different than 28°C and 32°C averaged (p-value < 0.05, Chi-Square Test with Yates Correction). Infection in the fluctuating EIT group appears to be lower than 28°C and 32°C; most closely resembling infection rates between 25°C and 28°C EITs or 32°C and 35°C EITs (Fig 1). Mosquitoes experiencing fluctuating EITs also had significantly lower dissemination and transmission (p-value <0.05, Chi-Square Test with Yates Correction) compared to a mean of the standard laboratory colony EIT of 28°C, which is used for most VC studies, and an EIT of 32°C.

### Intrahost genetic diversity

**Fig 2:**
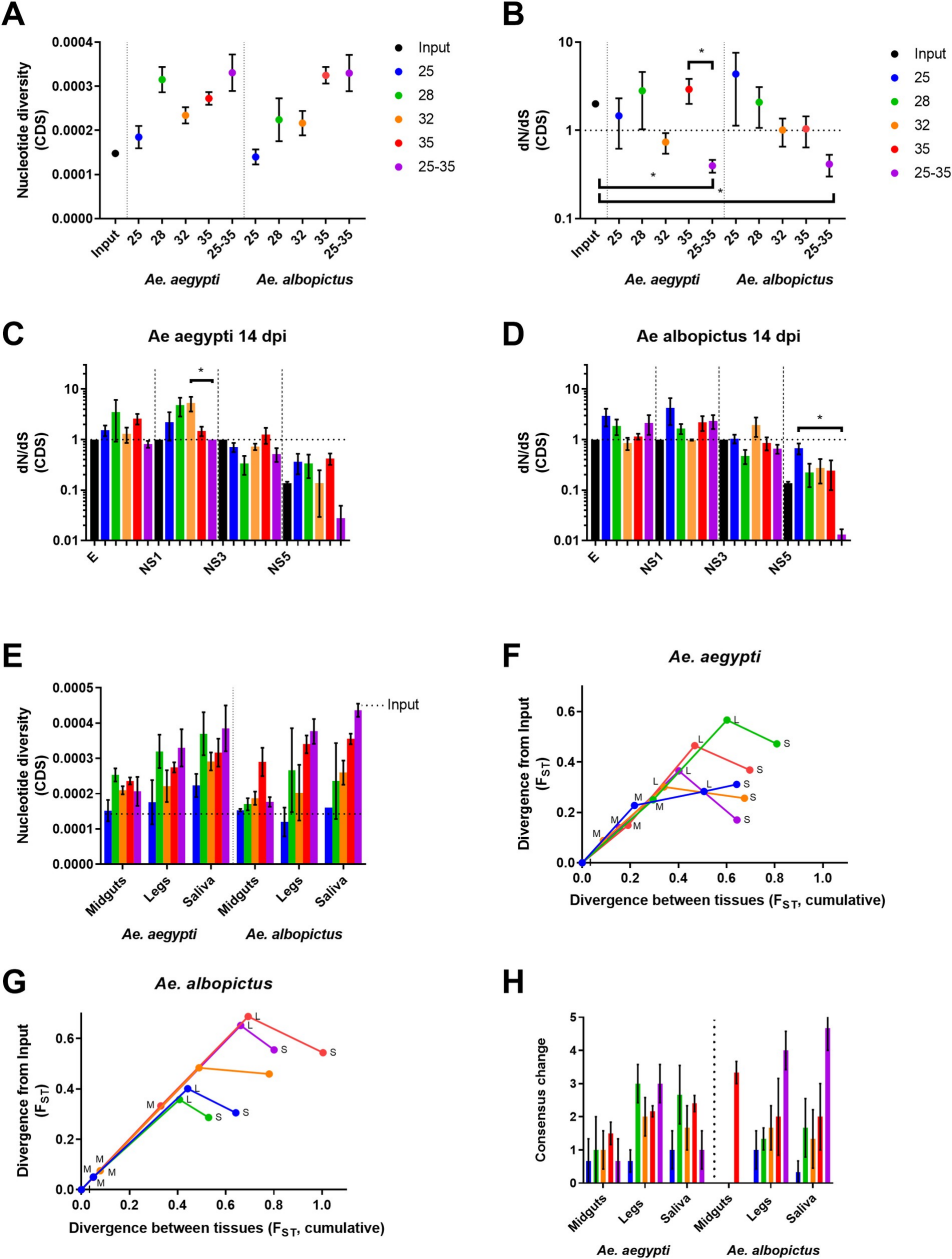
Extrinsic incubation temperature alters virus diversification, selection and divergence during mosquito infection. ZIKV population diversity at varying EITs was determined using measures of nucleotide diversity (A) and natural selection *(d*_*N*_*/d*_*S*_) (B). *d*_*N*_*/d*_*S*_ was also examined by virus coding region for virus that replicated in *Ae*. *aegypti* (C), and *Ae*. *albopictus* (D). Measured results for E, NS1, NS3, and NS5 protein coding regions are shown. *d*_*N*_*/d*_*S*_ was near 1 for E, prM NS2A, NS2B, NS4A, NS4B (not shown). Nucleotide diversity (E) and divergence (F-G) were determined for ZIKV in different mosquito compartments. Divergence from input population (y-axis) and cumulative divergence between tissues (x-axis) (F-G) is presented for Midguts (M), legs (L), and Saliva (S). Consensus change counts also are presented (H) by EIT and mosquito species, alongside majority variants accumulated (H), as markers of population diversity. Significance was tested using the Kruskal-Wallis test with Dunn’s correction (B-D, p < 0.05). Figures present the mean and SEM.

Consensus-level changes were rarely observed in ZIKV in these experiments. Statistical modeling revealed no significant factors associated with consensus level changes to the ZIKV genome (S1 and S2 Tables). We therefore assessed the effect of EIT and mosquito species on ZIKV mutational diversity at the intrahost level by sequencing midgut, legs, and saliva that were harvested from both *Ae*. *aegypti* and *Ae*. *albopictus* during VC experiments. The virus was collected from three mosquito replicates from each mosquito species for each EIT group at 14 days post exposure. Nucleotide diversity across the coding sequence was lowest in mosquitoes held at 25°C and highest in mosquitoes held at 35°C or under a fluctuating EIT regime (Fig 2A). This is supported by the best fit GLM

$$nucleotide\;diversity∼exp\;[\beta_{1}(dpi)+\beta_{2}(species)+{\beta_{3}(tissue)+\beta }_{4}(temp)]$$

which includes a significant effect of temperature on nucleotide diversity (S2 Table). Notably, the model does not include a unimodal temperature effect on nucleotide diversity and no interactions of fixed effects. Fluctuating EIT consistently produced the highest levels of nucleotide diversity in both mosquito species. However, the EIT of 28°C in *Ae*. *aegypti* and an EIT of 35°C in *Ae*. *albopictus* were second highest, suggesting a specific effect of EIT (Fig 2A). Selection acting upon viral genomes was assessed by estimating *d*_*N*_*/d*_*S*_ across the coding sequence. A *d*_*N*_*/d*_*S*_ > 1 suggests positive selection, *d*_*N*_*/d*_*S*_ < 1 suggests purifying selection, and *d*_*N*_*/d*_*S*_ of 1 suggest neutral evolution. In both *Aedes* species, *d*_*N*_*/d*_*S*_ was significantly lower in ZIKV from fluctuating EIT-exposed mosquitoes compared to those held at constant EITs, and virus from these mosquitoes was unique in having *d*_*N*_*/d*_*S*_ much lower than 1 (Fig 2B). Similar levels of richness, complexity, and divergence were observed in all mosquitoes and EIT groups (not shown).

To assess coding region-specific signatures of selection, we analyzed *d*_*N*_*/d*_*S*_ for each viral protein coding sequence independently. In both mosquito species exposed to fluctuating EITs, *d*_*N*_*/d*_*S*_ was much lower than 1, indicating purifying selection within the NS5 coding sequence (Fig 2C and 2D mean *d*_*N*_*/d*_*S*_ 0.027 in *Ae*. *aegypti* and 0.0132 in *Ae*. *albopictus*). E and NS1 coding sequences had *d*_*N*_*/d*_*S*_ greater than 1 when EITs were constant, with a range of mean *d*_*N*_*/d*_*S*_ in *Ae*. *aegypti* of 1.298 at 32°C in E to 5.325 at 32°C in NS1. *Ae*. *albopictus* had a mean low *d*_*N*_*/d*_*S*_ of 1.154 at 35°C in E and a high of 4.267 at 25°C in NS1, the exception being 32°C in *Ae*. *albopictus* where *d*_*N*_*/d*_*S*_ was 0.0853 (E) and 0.9804 (NS1) respectively (Fig 2C and 2D).

Since arboviruses encounter multiple replication environments and barriers during systemic mosquito infection, we next assessed intrahost population diversity in the midguts, legs, and saliva of mosquitoes held at varying EITs (Fig 2E–2H). Across EITs, nucleotide diversity in midgut infection tended to be lower than nucleotide diversity in leg infection (S2 Table). In all tissue compartments of both species tested, nucleotide diversity tended to increase with increasing EIT (Fig 2E and S2 Table), with fluctuating EIT-exposed mosquitoes having lower diversity during midgut infection but increased genetic diversity during systemic infection that resulted in the highest levels of nucleotide diversity in leg and saliva-associated virus (Fig 2E). Analysis of the fixation index (F_ST_), during systemic infection revealed EIT- and species-specific patterns of divergence from the input population (Fig 2F and 2G). Generally, ZIKV diverged more in the midguts of *Ae*. *aegypti* than *Ae*. *albopictus*. The 28°C EIT group diverged more than any other EIT group in *Ae*. *aegypti* (Fig 2F). Conversely, in *Ae*. *albopictus*, exposure to higher EIT of 32°C and 35°C promoted divergence. In both species, divergence was greatest when the population disseminated from the midgut to the legs and divergence was lowest from legs to saliva. These data provide evidence that divergence from the founding population was increased in the midgut and legs of both species and reduced as virus moved from legs to saliva, likely due to stochastic reductions in the complexity of the virus population coupled with retention of the most common virus genotypes from the prior compartment. Temperature also seemed to be associated with the number of consensus changes to the ZIKV sequence (Fig 2H), although this relationship was not statistically significant.

### Intrahost selective pressures

**Fig 3:**
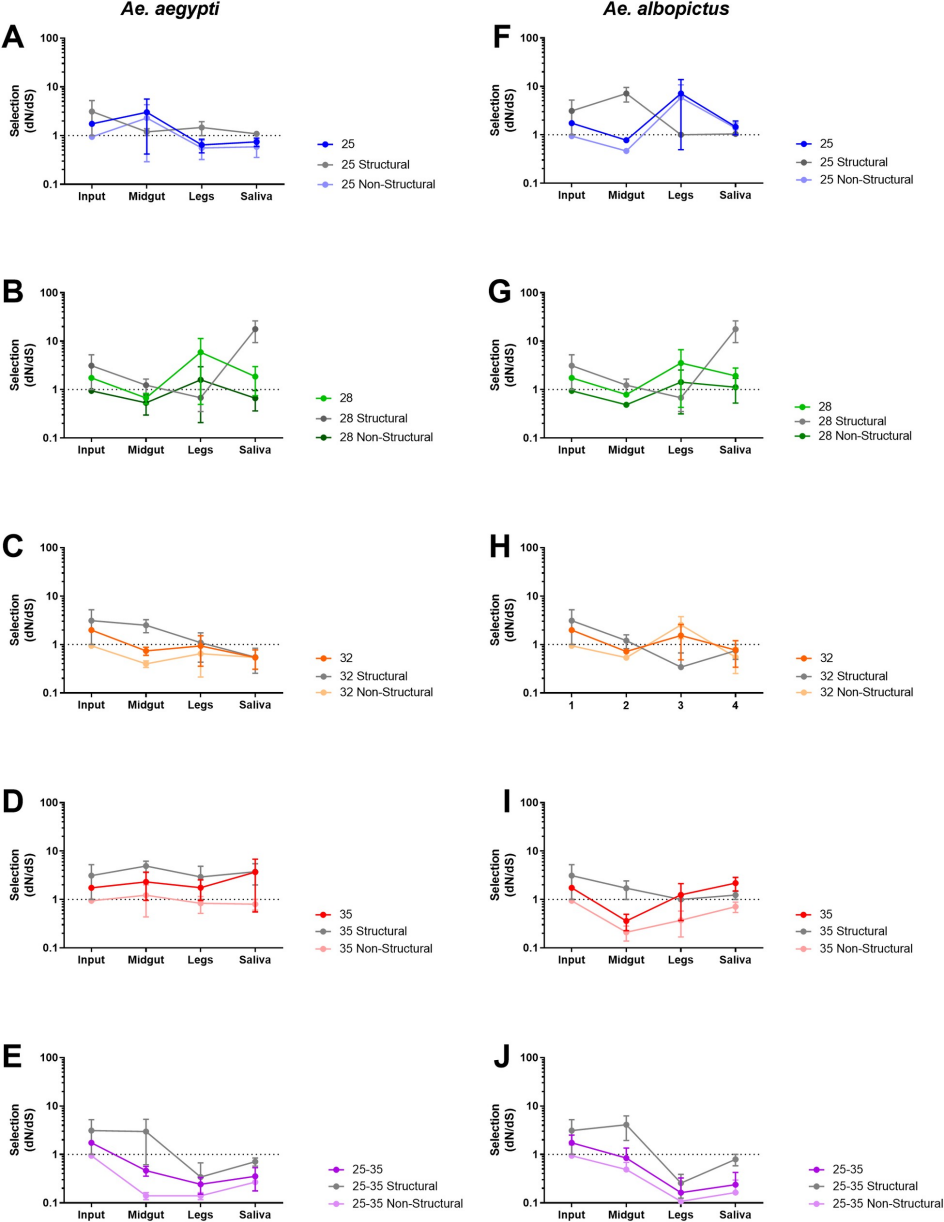
Fluctuating extrinsic incubation temperatures impose purifying selection on ZIKV during systemic mosquito infection. *d*_*N*_*/d*_*S*_ (mean with SEM) for ZIKV CDS (closed circles), structural sequence (Boxes), and non-structural sequence (open circles), at indicated EITs including fluctuating EITs, in *Ae*. *aegypti* (A-E) and *Ae*. *albopictus* (F-J).

Since ZIKV population diversity is influenced by the tissues of origin, as well as the constant and fluctuating EIT, we assessed *d*_*N*_*/d*_*S*_ in systemic infection for each EIT group across the entire CDS, and for the structural and nonstructural regions independently (Fig 3 and S4 Table). Our input population had a *d*_*N*_*/d*_*S*_ ratio of 1.75 for the CDS, 3.11 for the structural regions, and 0.95 for the non-structural regions. This suggests that the structural regions of our input population were under positive selection (*d*_*N*_*/d*_*S*_ greater than 1) during its propagation and preparation, whereas the non-structural regions were not. Interestingly, when ZIKV was exposed to fluctuating EITs, it presented strong purifying selection (*d*_*N*_*/d*_*S*_ less than 1) in both *Aedes* species (Fig 3E and 3J), whereas all constant EITs caused ZIKV *d*_*N*_*/d*_*S*_ to be generally near or above 1 (Fig 3). In the saliva, 25°C and 32°C EIT groups had a *d*_*N*_*/d*_*S*_ that neared 1, decreasing from the input in both *Aedes* species (Fig 3A, 3C, 3F, and 3H). Conversely, 28°C and 35°C EIT groups maintained or increased *d*_*N*_*/d*_*S*_ when compared to input (Fig 3B, 3D, 3G, and 3I).

### Intrahost extrinsic incubation temperature impacts on ZIKV variant frequency

**Fig 4:**
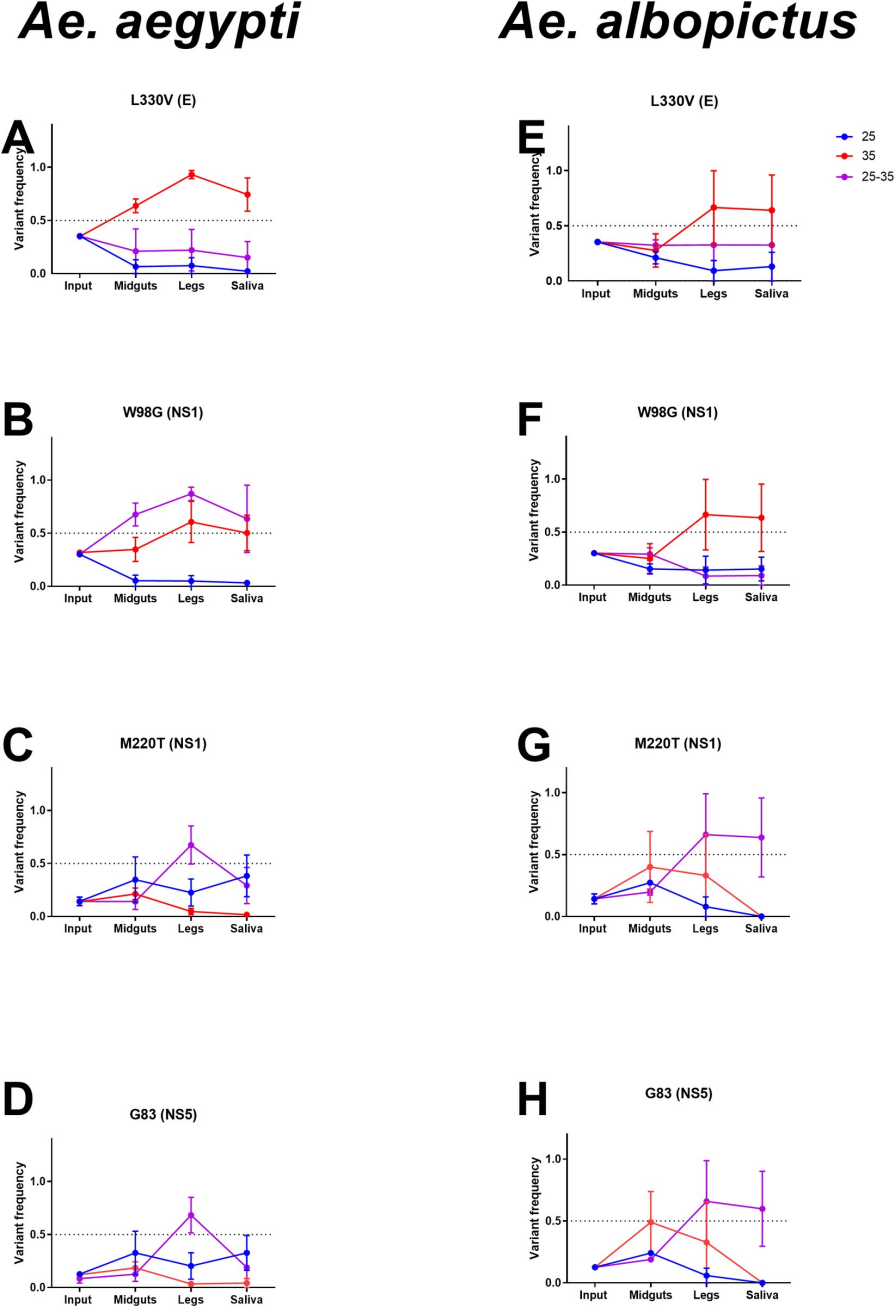
EIT and species control ZIKV variant frequency during systemic infection. Frequencies of L330V E (A & E), W98G NS1 (B & F), M220T NS1 (C & G), and G83 NS5 (D & H) in input, midgut, legs and saliva shown at 25, 35 and fluctuating 25–35°C in *Ae*. *aegypti* (A-D) and *Ae*. *albopictus* (E-H). Mean and SEM of competitions from three biological replicates shown.

Although relatively few consensus level changes occurred in ZIKV after replication in mosquitoes, we identified a handful of changes to the ZIKV genome that occurred independently in several mosquito tissues (Table 1). Of these consensus changes, 3 nonsynonymous and 1 synonymous SNVs were found in both *Ae*. *aegypti* and *Ae*. *albopictus* (L330V E, W98G NS1, M220T NS1, and G83 NS5) samples. The remaining 4 consensus changes were comprised of 1 non-synonymous mutation (T315I E) unique to *Ae*. *aegypti* and 3 mutations unique to *Ae*. *albopictus*: 1 non-synonymous mutation and 2 synonymous mutations (K146E NS1, I94 NS2A and F682 NS5). All were present as minority variants in the input virus population, and three have been documented in ZIKV genomic epidemiologic studies. (Table 1). These consensus changes tended to rise or fall in frequency in a species- and EIT-dependent manner (Fig 4). In general, high and/or fluctuating EIT tended to drive variants to higher frequency in the population, while at the low EIT (25°C) variants tended to remain closer to their input frequency. The frequency of the synonymous variant G83 in NS5 fluctuated in frequency similarly to M220T, suggesting linkage on the viral genome.

**Table 1:** Multiple ZIKV variants found in across biological samples and EITs go to consensus. ^a^ Variant frequency found in the stock input ZIKV population. ^b^ The percent sequence identity observed in nature when aligned to 150 complete ZIKV genomes. ^c^ Extrinsic incubation EITs at which each intrahost SNV (iSNV) was observed, ^d^ and tissues that each iSNV was observed. Black = both species, Blue = Ae. aegypti only, Red = Ae. albopictus only. (M) midguts; (L) legs; (S) saliva.

Species	Coding Sequence	AA	Input Freqa	Freq found in Natureb	Temperature Obs.^c^	Tissue Obs.^d^
***Aedes spp*.**	E	L330V	0.35	100%	28, 32, 35, 25–35	M, L, S
	NS1	W98G	0.30	0%	28, 32, 35, 25–35	M, L, S
	NS1	M220T	0.14	0%	25,28, 32, 35, 25–35	M, L, S
	NS5	*G83*	0.13	0%	25,28, 32, 35, 25–35	M, L, S
***Ae*. *aegypti***	E	T315I	0.05	0%	28,35	M,L,S
***Ae*. *albopictus***	NS1	K146E	0.01	2%	25,28,32,25–35	L,S
	NS2A	*I94*	0.03	0.7%	35,25–35	M,L,S
	NS5	*F682*	0.03	0%	35,25–35	M,L,S

### In-vivo competitive fitness

**Fig 5:**
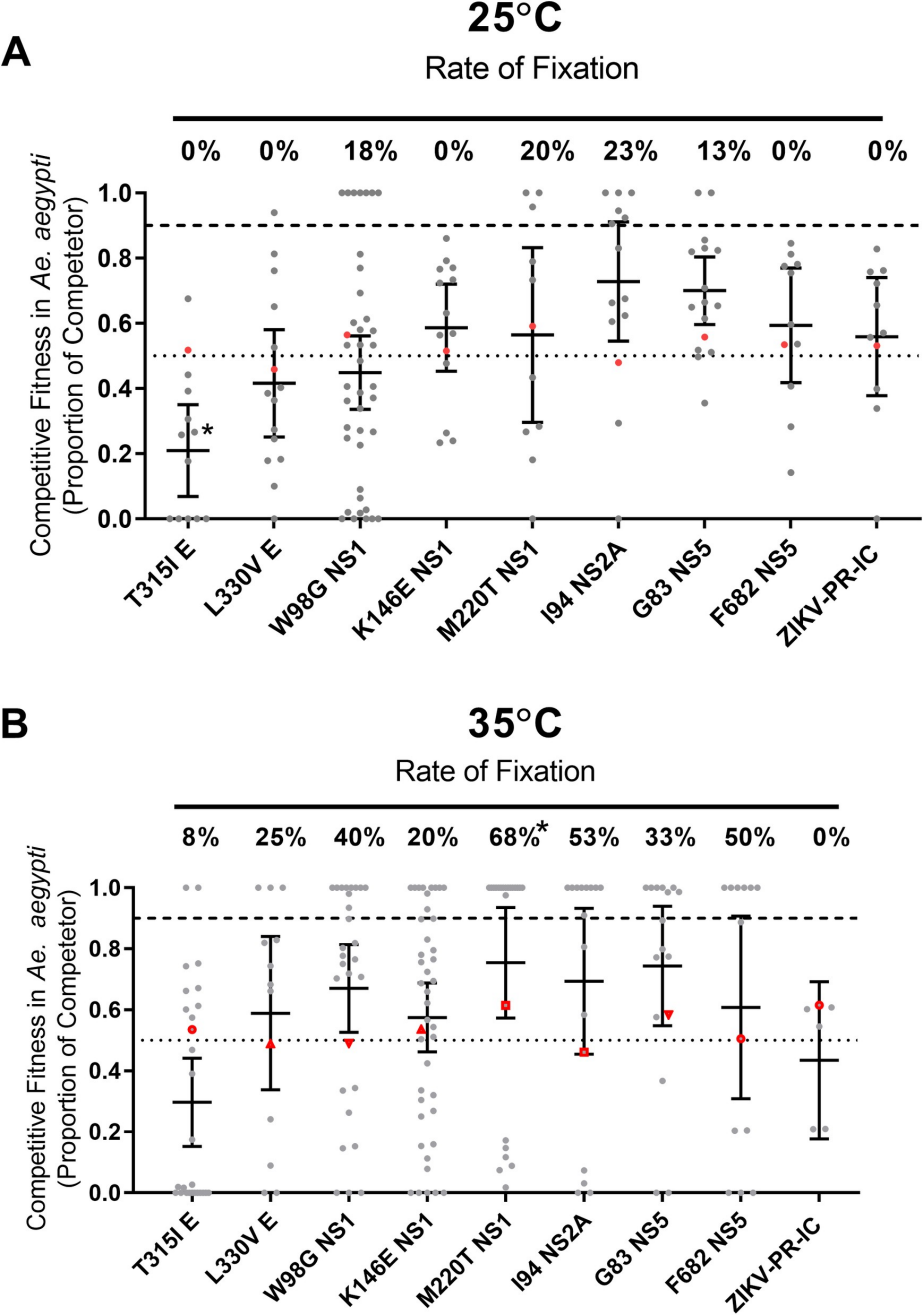
High extrinsic incubation temperature increases variant fixation in Aedes aegypti. Indicated mutations were engineered into a ZIKV-PR-IC and mixed with a ZIKV-REF virus. The proportion of each competitor (grey, mean with 95% CI, *p-value < 0.05 compared with ZIKV-PR-IC, Kruskal-Wallis and Dunn’s) and rate of fixation (*p-value < 0.05 compared with ZIKV-PR-IC, Two-tailed Fisher’s exact test) was determined from mosquito bodies at 14-dpi for *Ae*. *aegypti* mosquitoes held at constant EIT’s 25°C (A) & 35°C (B). Fixation indicates that 100% of the sequenced nucleotides were from the competitor virus. Initial viral inoculum (ratio of competitor virus to reference) is shown in red symbols.

We then compared engineered ZIKV mutants containing the eight consensus-changing mutations that arose during systemic infection (Table 1) in mosquitoes under low (25°C) and high (35°C) EITs (Fig 5) to assess whether the fitness of these variants may be EIT-dependent. Control competitions with marked and unmarked clones of the PRVABC59 virus were unremarkable, with no significant changes in test to reference virus detected at either EIT (Fig 5 and S5 Table).

Several mutants tended to rise in frequency at both 25°C and 35°C, with the overall rate of fixation (mutants that reached 100% frequency as measured by our assay) higher in mosquitoes held at 35°C (Fig 5, p< 0.001, Two-tailed Fisher’s exact test). The fitness implications of these mutants tended to be variable. For example, in orally exposed *Ae*. *aegypti* bodies, the clone bearing the NS1 M220T mutant had a significant (Fig 5A, p<0.05, Kruskal-Wallis and Dunn’s) fitness advantage 14 days after blood feeding compared to wildtype. Conversely, the envelope T315I mutant had significantly decreased (p<0.05, Kruskal-Wallis and Dunn’s) fitness compared to ZIKV-PR-IC at 25°C.

## Discussion

### Extrinsic incubation temperature alters vector competence

Vector competence is largely determined by barriers to infection and escape from mosquito midgut and salivary glands [7,40], and varies among naturally occurring mosquito populations [22]. Our results support the extensive existing literature [11,12,41] that EIT controls infection and escape mechanisms, resulting in a unimodal distribution of VC values: Extreme low 25°C and high 35°C EITs were associated with the lowest VC, while moderate EIT of 28°C and 32°C had peak VC (Fig 1). These results agree with previous studies [11] and mechanistic models predicting the ZIKV thermal optimum of 29°C for *Ae*. *aegypti* [11,42]. However, the VC of mosquitoes held at fluctuating EITs were consistently lower than the mean of moderate EITs 28°C and 32°C despite having a similar mean EIT. The reasons for this are unlikely to be caused by direct effects of temperature on virus replication because ZIKV readily undergoes replication in vertebrates that commonly maintain temperatures of 37°C, higher than any temperature tested here. It seems more likely that the depressed VC observed at higher temps is due to indirect alterations to some aspect of the mosquito environment. In addition, the timing of temperature changes resulted in mosquitoes being held at temperature extremes for much longer than being held near the mean/optimum temperature. Additionally, our study does not capture behavioral and physiological adaptations of mosquitoes to high or low EITs that may occur in nature. These could include seeking cooler resting sites or seeking shelter; or adaptations that could occur due to natural selection to an environment that warms over several decades. Nonetheless, our data clearly demonstrate that rapidly fluctuating EITs negatively influence mosquito vector competence for ZIKV compared to optimal constant EITs (Fig 1 and S3 Table).

### Extrinsic incubation temperature and vector species alter the selective environment within mosquitoes

Our data on intrahost population structure during systemic infection provides several novel insights into how EIT alters the selective environment in epidemiologically relevant arbovirus vectors. First, our data demonstrate that, in general, increasing EIT leads to increases in nucleotide diversity (Fig 2A). This may simply reflect faster virus replication at higher EITs, with additional rounds of replication introducing additional mutations in a somewhat clocklike fashion. Alternatively, it may be that this increase in nucleotide diversity reflects the strength of fixation at higher EITs (Fig 5). The exception to this is mosquitoes that were held at 28°C. Mosquitoes held at fluctuating EITs, as above, were somewhat atypical in that they tended to have higher levels of nucleotide diversity than one would expect given the distribution of diversity observed in mosquitoes held at constant EIT. As in the case of VC, fluctuating EITs are distinct in their impacts on virus-host interactions when compared to constant EITs and thus the impact and strength of selection is also distinct.

Analysis of synonymous and nonsynonymous variation at the intrahost level revealed that purifying selection was strongest in mosquitoes held at fluctuating EITs (Figs 2B–2D and 3E and 3J). This observation provides an important counterpoint to several previous studies that have documented a relaxation of purifying selection in flaviviruses that are undergoing replication in mosquitoes [43]. Data from mosquitoes held at constant EITs supports these prior findings: *d*_*N*_*/d*_*S*_ was generally near or above 1. In some cases, (e.g. 28°C and 35°C) positive selection was detected within the E and NS1 coding regions. Positive selection observed at 35°C seems likely to be due to virus adaption to some component of the mosquito stress response such has heat shock protein [44,45]. The mechanisms that give rise to the signal of positive selection at 28°C are not clear but may be due to a bottleneck that by chance resulted in a genome with one or more nonsynonymous variants that rose in frequency due to this stochastic event rather than natural selection. The strong purifying selection observed in mosquitoes held at fluctuating EITs (Fig 3E and 3J) is detectable mainly in nonstructural coding sequences, and principally in NS5 (Fig 2C and 2D). This seems logical given the role of NS5-encoded proteins in virus replication [46] and highlights the requirement for replicase functionality across a wide temperature gradient. Indeed, our data suggest that ZIKV NS5 is adapted to temperature variation rather than to any individual temperature. The *d*_*N*_*/d*_*S*_ in NS5 of the input population also was below 1, indicating that this portion of the genome was likely already under purifying selection in the input population. NS1 of mosquito-borne flaviviruses contributes to pathogenesis [47] and mosquito infectivity [48]. Thus, the signal of positive selection observed in these studies may be due to its role in establishing infection of the mosquito midgut, which was required in these studies.

Analysis of consensus sequences taken from different mosquito tissues allowed us to identify the impacts of virus dissemination and physiological barriers on ZIKV sequences during systemic infection. Generally, our data demonstrate a positive correlation between constant EIT and nucleotide diversity (Fig 2E). As the virus disseminates into saliva, nucleotide diversity increases both with EIT and in a stepwise manner as new tissue compartments are infected. These findings indicate that at higher EITs, more consensus variants are generated. The impact of fluctuating EITs differed between two species, with most new consensus variants generated at fluctuating EITs by the less-efficient vector *Ae*. *albopictus*. These observations demonstrate that temperature gradients and fluctuations, and virus transmission by new, perhaps less efficient vector species, may result in positive selection during mosquito infection, and are supported by analyses of divergence that incorporate both intra- and interhost variation. Therefore, migration of arboviruses such as ZIKV into new environments containing unexplored vectors represents an opportunity for the emergence of new virus variants.

### Increased extrinsic incubation temperatures drive viral variant fixation in mosquitoes

We identified eight consensus mutations (5 non-synonymous and 3 synonymous) in multiple mosquitoes during the course of this study. These all were present in the input population at low frequencies (0.01–0.35, Table 1) and rose in frequency during mosquito infection. Four consensus changes were found in both *Aedes* species and in mosquitoes held under most EIT regimes. L330V E (S1A Fig) is within domain III of the envelope protein and plays a role in host cell receptor binding and entry [49]. W98G NS1 (S1B Fig) is a surface exposed aromatic to aliphatic amino acid change on the wing section of NS1, which contributes to cellular membrane association [50]. M220T NS1 (S1C Fig) replaces a sulfur containing side group with a hydroxylic side group and is located on the loop surface the NS1 172–352 homodimer [51]. G83 NS5 (S1D Fig) is a synonymous mutation found in the middle of the coding sequence for the NS5 methyltransferase domain active site. With the exception of L330V E, which appears to be a revertant to that observed in nature [52](Table 1), most of these substitutions were not observed in our alignment of naturally occurring ZIKV mutations.

During systemic mosquito infection (Fig 4), none of these mutations increased significantly in frequency at 25°C. Mosquito exposure to 35°C or fluctuating EITs cause some of these mutations to rise in frequency, sometimes in a species-dependent manner, again highlighting the EIT-dependence of variant frequencies in our studies.

To assess the fitness implications of these and other mutations that were repeatedly detected in mosquitoes, we engineered individual mutations into a ZIKV infectious clone and conducted *in vivo* competition studies at 25° and 35°C. The most notable finding from these studies is that higher EITs tended to favor frequently detected mutations, which is consistent with our data on variant frequencies, consensus level changes and the strength of purifying selection. Accordingly, we conclude that the variants we examined are more likely to reach high frequency at higher EIT.

Although the fluctuating temperatures used in this study more accurately mimic natural conditions, they do not do so perfectly: the change in temperature under our experimental conditions occurs faster (<30 minutes) than in nature. Similarly, the fluctuating EIT group does not account for microclimates in which mosquito vectors may be found. Additionally, arbovirus vector competence and population diversity are directly impacted by specific host species and pathogen strains [53]. We aimed to account for this effect by using both *Ae*. *aegypti* and *Ae*. *albopictus* as vectors to determine EIT impacts of ZIKV VC and population dynamics. Last, we use *d*_*N*_*/d*_*S*_ to cautiously estimate the strength of purifying selection. Since we are accounting for all non-synonymous SNVs and not just consensus SNVs, we are likely to capture transient mutations awaiting selection rather than fixed mutations in the consensus. Unfortunately, due to the low number of consensus changes observed in the sequencing data, this was a limitation of the study.

The work presented here was designed to address the hypothesis that EIT influences not only the efficiency with which mosquitoes transmit arboviruses (which has been well established for decades) but that it also influences virus evolutionary dynamics. The lack of quantitative data on how EIT effects arbovirus mutational diversity and selective forces within mosquitoes is a critical shortcoming in the literature. Data presented here provides some novel insights into this. The most significant findings reported here are that EIT influences the rate of fixation of novel variants in virus populations. This suggests that as global temperatures rise, new arbovirus variants may emerge more rapidly with clear health implications.

A second key finding is that fluctuating EITs impose heretofore undetected purifying selection on virus populations as they pass through mosquitoes. This finding was not predicted but was consistent in both mosquito species examined. We suspect that this finding is related to both (a) increased constraints imposed by the requirement that the virus replicase acts efficiently across a ten-degree Celsius temperature range and (b) the inability of potentially temperature-specific adaptive mutations to rise in frequency. Our data on individual mutations in various mosquito tissues supports this. Moreover, this work collectively highlights the significance of environmental temperature (and changes in temperature) on the evolutionary biology of the mosquito-virus interaction. It also indicates that studies of arbovirus-vector interactions conducted using multiple EITs, including fluctuating EIT cycles, may capture subtle yet significant evolutionary forces that act on viruses during mosquito infection.

## Methods

### Cells and virus

African Green Monkey kidney cells (Vero; ATCC CCL-81) were maintained at 37°C and 5% CO_2_ in Dulbecco’s modified Eagle’s medium (DMEM) supplemented with 10% fetal bovine serum (FBS) and 1% penicillin-streptomycin (Pen-Strep). Zika virus strain PRVABC59 (ZIKV-PRVABC59; GenBank # KU501215) obtained from the Center for Disease Control and Prevention branch in Fort Collins, CO was originally isolated from the sera of a patient returning from travel to Puerto Rico in December 2015. The virus was isolated on Vero cells and a 4^th^ passage frozen at -80 was used for all *in vivo* and *in vitro* experiments. ZIKV-PRVABC59 infectious clone (ZIKV-PR-IC) served as a backbone for the reverse genetic platform developed by our lab [53] to introduce all point mutations. ZIKV-REF was designed using the aforementioned reverse genetic platform, incorporating 5 synonymous mutations amino acid 108-arganie and 109-serine of the prM protein coding sequence. The ZIKV-PR-IC sequence nucleotides were changed from ZIKV-PR-IC 795-CGG TCG-800 to ZIKV-REF 795-AGA AGT-200.

### Mosquitoes

*Ae*. *aegypti* colonies for this study were established from individuals collected in Poza Rica, Mexico [54] and used at F13-F18 generation. A lab adapted colony (greater than 50 generations) of *Ae*. *albopictus* was established from individuals collected in Florida, USA; the colony was provided by the Centers for Diseases Control and Prevention (CDC-Fort Collins, CO, USA) in 2010. Mosquitoes were reared and maintained at 27–28°C and 70–80% relative humidity with a 12:12 L:D photoperiod. Water and sucrose were provided ad libitum.

### Infection of *Aedes* mosquitoes and sample collection

Adult mosquitoes used for experiments were 3–7 days post-eclosion. Mosquitoes were provided a bloodmeal containing calf blood mixed 1:1 with ZIKV-PRVABC59 (1E7 PFU/mL) using a water jacketed glass membrane feeder. Engorged female mosquitoes were sorted into cartons and housed at 25°C, 28°C, 32°C, 35°C constant EITs or alternating between 25°C—35°C to simulate diurnal fluctuating conditions, with 70–80% relative humidity and 12:12 L:D photoperiod. Mosquitoes were cold anesthetized in preparation for dissociations. Mosquito midguts, legs/wings, and saliva from the first batch of mosquitoes were collected after 7- and 14-days post-feed for NGS processing. Mosquito carcass, legs/wings and saliva from the second batch of mosquitoes were collected at 3, 5, 7, 10, and 14 days post-feed for assessing systemic infecting dynamics. Tissues represent infection (midgut), dissemination (legs), and transmission (saliva). Tissues were removed using forceps cleaned with 70% ethanol between samples and were homogenized in 200 μl of mosquito diluent with a stainless-steel ball bearing using a Retsch Mixer Mill 400 at 24 Hz for 45 seconds, as previously described [55]. Saliva was collected by inserting mosquito mouthparts into capillary tubes containing mineral oil for 30 to 45 minutes. Saliva in oil was removed from the capillary tube by centrifugation into 100 μl of mosquito diluent for 5 minutes at >20,000 x g. All samples were stored at -80°C until manipulation.

### Plaque assay

ZIKV stocks and infectious bloodmeal were quantified by plaque assay on Vero cell cultures seeded in 12-well plates. Duplicate wells were infected with 0.2 ml aliquots from serial 10-fold dilutions of virus stocks and infectious blood meals in media (DMEM supplemented with 1% FBS and 1% penicillin/streptomycin), and virus was adsorbed for one hour by incubating at 37°C in 5% CO_2_. Following incubation, the inoculum was removed, and monolayers were overlaid with tragacanth-EMEM overlay containing 1x EMEM, 5x L-glutamine, sodium bicarbonate 3.75%, tragacanth 1.2%, gentamicin (25mg/ml), and Amphotericin B 40mL/L. Cells were incubated at 37°C in 5% CO_2_ for four days for plaque development. Cell monolayers then were stained with 1 mL of overlay containing a 20% ethanol and 0.1% crystal violet. Cells were incubated at room temperature for 30–60 minutes and then gently washed and plaques were counted. Plaque assays for 3, 5, 7, 10 and 14 days post infection (dpi) mosquitoes were performed similar to above with the following changes: 50 ul of homogenized midgut and leg tissues or 30 ul of saliva samples were added to Vero cultures in 24-well plates (final volume of 200 ul), and plaques were observed post processing.

### Viral RNA isolation

Viral RNA was extracted from 50 μl of either cell culture supernatant, homogenized mosquito tissues, or saliva-containing solution using the Mag-Bind Viral DNA/RNA 96 kit (Omega Bio-Tek) on the KingFisher Flex Magnetic Particle processor (Thermo Fisher Scientific). Nucleic acid extraction was performed as directed by the manufacturer and eluted in 50 μl nuclease-free water. Viral RNA was then quantified by qRT-PCR using the iTaq Universal Probes One-Step Kit (BIO-RAD) according to manufacturer’s protocol using forward primer (5’- CCGCTGCCCAACACAAG-3’), reverse primer (5’- CCACTAACGTTCTTTTGCAGACAT-3’), and FAM probe (5’- AGCCTACCTTGACAAGCAGTCAGACACTCAA-3’) sequences [56].

### Generation of ZIKV mutant clones

An infectious clone for ZIKV-PRVABC59 was used to generate mutants [53]. To engineer the point mutations (Table 1) into the ZIKV genome, the corresponding single nucleic acid substitution was introduced into the ZIKV-PR-IC using *in vivo* assembly cloning methods [57]. The infectious clone plasmids were linearized by restriction endonuclease digestion, PCR purified, and ligated with T4 DNA ligase. From the assembled fragments, capped T7 RNA transcripts were generated, and the resulting RNA was electroporated into Vero cells. Infectious virus was harvested when 50–75% cytopathic effects were observed (5–8 days post transfection). Viral supernatant was clarified by centrifugation and supplemented to a final concentration of 20% fetal bovine serum and 10 mM HEPES prior to freezing and storage as single use aliquots. Titer was measured by plaque assay on Vero cells. All stocks (both wildtype and infectious clone-derived viruses) were sequenced via sanger sequencing to verify complete genome sequence.

### Competition studies

Competitive fitness was determined largely as described in previous studies [58,59]. Competitions were conducted with orally infected *Ae*. *aegypti* (Poza Rica) mosquitoes. Three to seven-day old mosquitoes were offered a bloodmeal containing the 1:1 mixture of viruses (ZIKV-REF and ZIKV-clone of interest) at a concentration of 1 million PFU/mL and bodies were collected 14 days post blood feed. RNA was extracted as above, and amplicons were generated via qRT-PCR using iTaq Universal SYBR Green One-Step Kit (BIO-RAD) according to manufacture protocol. A locked nucleic acid (LNA) forward primer was used to ensure amplicon specificity. The forward LNA primer 5’-A+CTTGGGTTGTGTACGG-3’ and reverse primer 5’- GTTCCAAGACAACATCAACCCA-3’ were used to generate amplicons for Quantitative Sanger sequencing. Genotype fitness was analyzed using polySNP software [60] to measure the proportion of the five synonymous variants present in the ZIKV-REF sequence allowing us to compare the proportion of ZIKV-REF virus to competitor virus.

### Library preparation for next-generation sequencing

Virus was collected from three mosquito replicates from each mosquito species and EIT group, midgut, legs, and saliva were harvested and matched to mosquito for sequencing. Positive controls were generated in triplicate, each generated with 1 million genome equivalents of a 100% ZIKV PRVABC59 viral stock, a mixture of 90% ZIKV PRVABC59 and 10% ZIKV PA259359 (GenBank # KX156774.2), and a mixture of 99% ZIKV PRVABC59 and 1% ZIKV PA259359. The negative control was water (no template control, or NTC). Controls and sample RNA (10ul) was prepared for NGS using the Trio RNA-Seq Library Preparation Kit (NUGEN) per manufacturer standard protocol. Final libraries were pooled by tissue type and analyzed for size distribution using the Agilent High Sensitivity D1000 Screen Tape on the Agilent Tapestation 2200, final quantification was performed using the NEBNext Library Quant Kit for Illumina (NEB) according to manufacturer’s protocol. 150 nt paired-end reads were generated using the Illumina HiSeq4000 at Genewiz.

### NGS processing and data analysis

NGS data were analyzed using a workflow termed “RPG (RNA virus Population Genetics) Workflow”; this workflow was generated using Snakemake [61] and the workflow and related documentation can be found at https://bitbucket.org/murrieta/snakemake/src. Briefly, Read 1 and Read 2.fastq files from paired-end Illumina HiSeq 4000 data were trimmed for Illumina adaptors and quality trimming of phred scores < 30 from the 3’ and 5’ read ends using cutadapt [62]. The reads were then mapped to the ZIKV-PRVABC59 reference sequence (GenBank # KU501215) using MOSAIK [63], similar to that previously described [64]. Picard [65], Genome Analysis Toolkit (GATK) [66], and SAMtools [67] were used for variant calling preprocessing. SNV’s and inserts and deletions (INDELS) we called using LoFreq [68] with the—call-indels command; otherwise, all settings were default. Consensus sequences were generated using the.vcf files generated above and VCFtools [69]. NTC had less than 0.02% of reads mapping to ZIKV and an average of <8x coverage across the genome indicating little to no contamination, sequencing bleed through, or index hopping (S6 Table). Only variants in the coding sequence (nt position 108–10379), with 100x coverage or greater and a cut off of 0.01 frequency were used for analysis to account for low coverage (reads per genome position) in the 3’ and 5’ untranslated regions (S6 Table, S2 Fig).

Data analysis was performed using custom Python and R code integrated into the RPG Workflow. Using .vcf files generated by LoFreq and .depth files generated by GATK DepthOfCoverage command, the workflow generates .csv files that provides sequencing coverage across the CDS, Shannon entropy, richness, nucleotide diversity, *d*_*N*_*/d*_*S*_, and F_ST_ (compared to input population) of a specified locus. Additionally, amino acid changes, synonymous (S) and non-synonymous (NS) changes, and Shannon entropy are reported by variant positions. The same scripts are called manually outside of the RPG Workflow to perform the above analysis on specific protein coding regions or to compare divergence of populations other than the input.

### Genetic diversity

All genetic diversity calculations were incorporated into Python and R code located at https://bitbucket.org/murrieta/snakemake/src/master/scripts/. In short, richness was calculated by the sum of the intrahost SNV (iSNV) sites detected in the CDS in each population. Diversity was calculated by the sum of the iSNV frequencies per coding sequence. Complexity was calculated using Shannon entropy (*S*) which was calculated for each intrahost population (*i*) using the iSNV frequency (*p*) at each nucleotide position (*s*):

$$\left(1)\;S_{i.s}=-(p_{s}({log}_{2}p_{s})+(1-p_{s}){log}_{2}(1-p_{s})\right)$$

The mean S from all sites s is used to estimate the mutant spectra complexity. Divergence was calculated using F_ST_ to estimate genetic divergence between two viral populations as described previously [64]

### Selection

Intrahost selection was estimated by the ratio of nonsynonymous (*d*_*N*_) to synonymous (*d*_*S*_) SNVs per site (*d*_*N*_*/d*_*S*_) using the Jukes-Cantor formula as previously described [62], and incorporated into custom Python code found at https://bitbucket.org/murrieta/snakemake/src/master/scripts/.

$$(2)\;d_{N}=\frac{-3(lnln(1-(\frac{4p_{n}}{3})))}{4}$$

and

$$(3)\;d_{S}=\frac{-3(lnln(1-(\frac{4p_{s}}{3})))}{4}$$

where *p*_*n*_ equals *Nd* (sum of the nonsynonymous iSNV and iLV frequencies accepted by the variant caller) divided by the number of nonsynonymous sites and *p*_*s*_ equals *Sd* (sum of the synonymous iSNVs) divided by the number of synonymous sites. DnaSP was used to determine the number of nonsynonymous (7822.83) and synonymous (2446.17) sites from the ancestral input ZIKV consensus sequence using the Nei-Gojorori method [69,70]. When no synonymous SNVs sites were present in replicates, *d*_*N*_*/d*_*S*_ was set to 1, and no nonsynonymous SNV’s *d*_*N*_*/d*_*S*_ was set to 0.

### Statistical analysis

All analyses were performed using GraphPad Prism (version 7.04) and R. Fisher’s exact test were used to determine significant difference in virus titers and viral loads. All other tests were done using Kruskal-Wallis with Dunn’s correction unless otherwise noted.

To evaluate the relationship between external factors and the infection dynamics of ZIKV, we examined the data with generalized linear model (GLM) analysis (R software, package stats). The predictors used include days post infection (dpi), temperature (scaled), species, and tissue type. The fluctuating daily temperature (25–35°C) was averaged to 30°C as the mean daily temperature for the purposes of this regression. We evaluated the impact of these variables on consensus changes, complexity, nucleotide diversity, richness and vector competence for each tissue type. As consensus changes and richness are counts of variants, the associated GLMs were compiled with Poisson distributions and log link functions. Complexity and nucleotide diversity are assumed to follow a normal distribution, so the GLM was compiled with a gaussian distribution and identity link function. Vector competence is presence or absence of virus in a particular tissue, as such a binomial distribution with a logit link function was used to build the GLM. The original model for measures of genetic diversity were built with the following structure (× indicates inclusion of all interactions between fixed effects):

$$response∼exp\;[\beta_{1}(dpi)\times \beta_{2}(species)\times \beta_{3}(tissue)\times \beta_{4}(temp)+{\beta_{5}(temp)}^{2}]$$

The original model for vector competence follows the structure:

$$response∼exp\;[\beta_{1}(dpi)\times \beta_{2}(species)\times \beta_{4}(temp)+{\beta_{5}(temp)}^{2}]$$

For each measure of genetic diversity and vector competence, we evaluated multiple models starting with a full model including all predictor interactions and a squared polynomial term for temperature. Each evaluation began with this full model, followed by reduction to the most parsimonious structure using Aikaike information criterion (AIC) values and deviance evaluations to ensure goodness of fit. The polynomial on temperature allows us to differentiate between the linear and quadratic effect of temperature.

A separate evaluation was done to compare the fluctuating EIT group to static EIT groups. Mean values were calculated for VC between EIT groups 28°C and 32°C to most accurately represent the optimal mean for static colony temperature, and these were compared with the VC values for the fluctuating EIT group (25–35°C). P-values (threshold < 0.05) were calculated using a Pearson’s Chi-Square Test of Independence with a Yates correction to prevent overestimation of statistical significance for small data. The null hypothesis for each Chi-Square test was that the vector competence of indicated combination of species, tissue, and days post infection is independent of static or fluctuating EIT (S3 Table). Comparisons with small sample sizes were also analyzed with a Fisher Exact Test for Count Data to account for the limitation of Chi-Square tests of data with small sample sizes. All Fisher Exact Test results were consistent with the Chi-Square results.

## Supporting information

S1 Table Model selection from generalized linear models (GLM). (DOCX)

S2 Table Results from parsimonious generalized linear models. (DOCX)

S3 Table Vector competence results. (XLSX)

S4 Table *Ae*. *aegypti* and *Ae*. *albopictus* loci d_N_/d_S_. (XLSX)

S5 Table *Ae*. *aegypti* in-vivo competitive fitness results. (XLSX)

S6 Table Sequencing summary for all biological samples. (XLSX)

S1 Fig Protein structure and location of Aedes consensus changes. (TIF)

S2 Fig Mean depth of coverage across the ZIKV CDS for 14 dpi *Ae*. *aegypti* and *Ae*. *albopictus*. (TIF)

S3 Fig Kaplan-Meier estimate of the survival of ZIKV exposed *Ae*. *albopictus* (A) and *Ae*. *aegypti* (B) at 5 different temperature groups (25°C, 28°C, 32°C, 35°C and 25°C-35°C).
(TIF)
