# Supplementary material for: Impact of extrinsic incubation temperature on natural selection during Zika virus infection of Aedes aegypti and Aedes albopictus
Source: PLoS Pathog. 2021 Nov 9;17(11):e1009433. doi: 10.1371/journal.ppat.1009433 (PMC8629396; doi:10.1371/journal.ppat.1009433)
Supplement: S1 Table — (DOCX) [file ppat.1009433.s001.docx]

| **Consensus Changes Model** | **AIC** | **DF** |
| --- | --- | --- |
| *Consensus Changes ~ dpi × s × t × T + T^2* | 479.08 | 138 |
| *Consensus Changes ~ dpi × s × t × T + T^2 - dpi:s:t:T* | 487.19 | 140 |
| *Consensus Changes ~ dpi × s × t × T + T^2* *- dpi:s:t:T - s:t:T - d:t:T - d:s:t - s:d:T* | 480.03 | 145 |
| ***Consensus Changes ~ dpi × s × t × T*** | **477.78** | **139** |
| *Consensus Changes ~ dpi × s × t × T - dpi:s:t:T* | 485.58 | 141 |
| **Richness Model** | **AIC** | **DF** |
| ***Richness ~ dpi × s × t × T + T^2*** | **1253.2** | **138** |
| *Richness ~ dpi × s × t × T* | 1286.1 | 139 |
| *Richness ~ dpi × s × t × T + T^2 - dpi:s:t:T* | 1265.4 | 140 |
| **Complexity Model** | **AIC** | **DF** |
| *Complexity ~ dpi × s × t × T + T^2* | -2224.3 | 138 |
| *Complexity ~ dpi × s × t × T + T^2 - dpi:s:t:T* | -2225 | 140 |
| *Complexity ~ dpi × s × t × T + T^2 - dpi:s:t:T - s:t:T* | -2228.7 | 142 |
| *Complexity ~ dpi × s × t × T + T^2 - dpi:s:t:T - s:t:T - dpi:t:T* | -2231.0 | 144 |
| ***Complexity ~ dpi + s + t + T + T^2 + dpi:s + dpi:t + s:t + dpi:T + s:T*** | **-2233.3** | **149** |
| **Nucleotide Diversity Model** | **AIC** | **DF** |
| *Nucleotide Diversity ~ dpi × s × t × T + T^2* | -2575.4 | 138 |
| *Nucleotide Diversity ~ dpi × s × t × T + T^2 - dpi:s:t:T* | -2578.2 | 140 |
| *Nucleotide Diversity ~ dpi × s × t × T + T^2 - dpi:s:t:T - s:t:T* | -2582.9 | 142 |
| *Nucleotide Diversity ~ dpi × s × t × T + T^2 - dpi:s:t:T - s:t:T - dpi:s:t* | -2586.3 | 144 |
| *Nucleotide Diversity ~ dpi × s × t × T + T^2 - dpi:s:t:T - s:t:T - dpi:s:t - dpi:t:T* | -2588.8 | 146 |
| *Nucleotide Diversity ~ dpi + s + t + T + T^2* | -2600.4 | 156 |
| ***Nucleotide Diversity ~ dpi + s + t + T*** | **-2601.2** | **157** |
| **Vector Competence - Midgut Model** | **AIC** | **DF** |
| ***Midgut Vector Competence ~ dpi × s × T + T^2*** | **398.08** | **53** |
| *Midgut Vector Competence ~ dpi × s × T* | 485.58 | 54 |
| *Midgut Vector Competence ~ dpi × s × T + T^2 - dpi:s:T* | 438.01 | 54 |
| **Vector Competence - Legs Model** | **AIC** | **DF** |
| ***Leg Vector Competence ~ dpi × s × T + T^2*** | **598.06** | **53** |
| *Leg Vector Competence ~ dpi × s × T* | 679.53 | 54 |
| *Leg Vector Competence ~ dpi × s × T + T^2 - dpi:s:T* | 630.85 | 54 |
| **Vector Competence - Saliva Model** | **AIC** | **DF** |
| *Saliva Vector Competence ~ dpi × s × T + T^2* | 326.86 | 30 |
| *Saliva Vector Competence ~ dpi × s × T* | 488.68 | 31 |
| *Saliva Vector Competence ~ dpi × s × T + T^2 - dpi:s:T* | 324.99 | 31 |
| ***Saliva Vector Competence ~ dpi + s + T + T^2 + dpi:T + s:T*** | **323.01** | **32** |

**S1 Table. Model selection from generalized linear models (GLM).**

T = temperature, dpi = days post infection, s = species, t = tissue

(×) indicates interactions between the fixed effects as denoted in the interactions column

(+) indicates only the given effect.
