# Supplementary material for: Impact of extrinsic incubation temperature on natural selection during Zika virus infection of Aedes aegypti and Aedes albopictus
Source: PLoS Pathog. 2021 Nov 9;17(11):e1009433. doi: 10.1371/journal.ppat.1009433 (PMC8629396; doi:10.1371/journal.ppat.1009433)
Supplement: S2 Table — (DOCX) [file ppat.1009433.s002.docx]

| **Consensus Changes** | | | |
| --- | --- | --- | --- |
| **Coefficient** | **Estimate** | **Std. Error** | **P-Value (* <0.05)** |
| (Intercept)  days  temp_scaled  speciesAl  tissueM  tissueS  days:temp_scaled days:speciesAl  temp_scaled:speciesAl days:tissueM  days:tissueS  temp_scaled:tissueM  temp_scaled:tissueS speciesAl:tissueM speciesAl:tissueS days:temp_scaled:speciesAl  days:temp_scaled:tissueM days:temp_scaled:tissueS days:speciesAl:tissueM days:speciesAl:tissueS temp_scaled:speciesAl:tissueM temp_scaled:speciesAl:tissueS days:temp_scaled:speciesAl:tissueM days:temp_scaled:speciesAl:tissueS | 0.578723452 0.012329894 0.171555089 0.022648043 -1.354834297 -1.824338514 -0.004329583 0.006353042  -0.203250850 0.041349226 0.126358113 1.279015327  1.721792910 23.725121125 1.073460235 0.023663924  -0.079661135 -0.119119684 -3.489763122 -0.080256043  -20.316312607 -1.022730734 2.768092592 0.066138955 | 4.065191e-01 3.548928e-02 4.008908e-01 5.702365e-01 8.532960e-01 1.153374e+00 3.428780e-02 5.047674e-02  5.674475e-01 7.131802e-02 8.772691e-02 8.088476e-01 1.168839e+00 1.624360e+03 1.463240e+00 5.008676e-02 6.703374e-02 8.797133e-02 2.320513e+02 1.136338e-01 1.189014e+03 1.892979e+00 1.698590e+02 1.428306e-01 | 0.1545602 0.7282714 0.6686987 0.9683188 0.1123393 0.1137087 0.8995168 0.8998421 0.7202053 0.5620586 0.1497663 0.1138138  0.1407297 0.9883466 0.4631812 0.6365995 0.2346862 0.1757128 0.9880013 0.4800209 0.9863675 0.5890069 0.9869979 0.6433224 |
| **Richness** | | | |
| **Coefficient** | **Estimate** | **Std. Error** | **P-Value (* <0.05)** |
| (Intercept)  days  speciesAl  tissueM  tissueS  temp_scaled I(temp_scaled^2)  days:speciesAl  days:tissueM  days:tissueS speciesAl:tissueM speciesAl:tissueS days:temp_scaled speciesAl:temp_scaled tissueM:temp_scaled tissueS:temp_scaled days:speciesAl:tissueM days:speciesAl:tissueS days:speciesAl:temp_scaled days:tissueM:temp_scaled days:tissueS:temp_scaled speciesAl:tissueM:temp_scaled speciesAl:tissueS:temp_scaled days:speciesAl:tissueM:temp_scaled days:speciesAl:tissueS:temp_scaled | 1.860666131 0.052469453 1.549381629 1.146179676 0.657061889 0.022847945 -0.129288774 -0.136728622 -0.045068049 -0.023969387 -1.505029643 -1.712163158 -0.004739153 -0.412454623 -0.883987706 0.784584041 0.146424910 0.132044772 0.040033584 0.080146392 -0.048680064 1.122027210 0.540739242 -0.118637446 -0.055848692 | 0.19481166 0.01633174 0.25468646 0.23678604 0.30627410 0.20235901 0.02214105 0.02318327 0.02016580 0.02477049 0.31577136 0.44354945 0.01672008 0.26701402 0.24334487 0.35704556 0.02839373 0.03695018 0.02443893 0.02042345 0.02787948 0.33134922 0.63172502 0.02982886 0.04977421 | 1.283231e-21  1.314801e-03 *  1.175969e-09 *  1.294665e-06 *  3.192572e-02 *  9.101035e-01  5.241262e-09 *  3.685393e-09 *  2.542557e-02 *  3.332148e-01  1.877324e-06 *  1.133219e-04 *  7.768390e-01  1.224206e-01  2.805211e-04 *  2.798947e-02 *  2.510108e-07 *  3.521208e-04 *  1.013994e-01  8.700653e-05 *  8.079545e-02  7.085798e-04 *  3.920130e-01  6.971093e-05 *  2.618451e-01 |
| **Complexity** | | | |
| **Coefficient** | **Estimate** | **Std. Error** | **P-Value (* <0.05)** |
| (Intercept)  days  speciesAl  tissueM  tissueS  temp_scaled  I(temp_scaled^2)  days:speciesAl  days:tissueM  days:tissueS  speciesAl:tissueM  speciesAl:tissueS  days:temp_scaled  speciesAl:temp_scaled | 4.690673e-04 -6.281784e-07 2.191887e-04 4.411965e-05 2.090634e-04 -1.864434e-04 -6.263477e-05 -2.304203e-05 1.928944e-05 7.206515e-06  6.912594e-05 -1.353393e-04 1.364964e-05 -2.293667e-05 | 1.214735e-04 1.038075e-05 1.346269e-04 1.488400e-04 1.920415e-04 6.993199e-05 2.066458e-05 1.128457e-05 1.258384e-05 1.537006e-05 8.812358e-05  1.020337e-04 5.668247e-06 3.934041e-05 | 0.0001675456 0.9518275304 0.1056121604 0.7673195556 0.2780718902 0.0085217423 * 0.0028748115 * 0.0429247054 *  0.1274274331 0.6398498496 0.4340387705  0.1867309243 0.0172585072 * 0.5607540232 |
| **Nucleotide Diversity** | | | |
| **Coefficient** | **Estimate** | **Std. Error** | **P-Value (* <0.05)** |
| (Intercept)  days  speciesAl  tissueM  tissueS  temp_scaled | 2.329738e-04 2.536778e-06 6.445484e-06 -6.320135e-05 3.008040e-05 2.306161e-05 | 10.0294806 1.3646371 0.5058933 -4.3485380 1.7959713 3.6187487 | 1.328780e-18 1.743198e-01 6.136412e-01 2.455556e-05 * 7.442204e-02 3.987451e-04 * |
| **Vector Competence - Midgut** | | | |
| **Coefficient** | **Estimate** | **Std. Error** | **P-Value (* <0.05)** |
| (Intercept)  days  speciesAl  temp_scaled  I(temp_scaled^2)  days:speciesAl  days:temp_scaled speciesAl:temp_scaled days:speciesAl:temp_scaled | 2.91088067 -0.06711249 -1.13820334 -0.89401048 -0.47533952 0.17446659 0.02278040 1.22825402 -0.17414547 | 0.19890055 0.01607541 0.33311162 0.16891574 0.04919162 0.03379190 0.01386703 0.28631861 0.02842941 | 1.683349e-48 2.981778e-05 * 6.334256e-04 * 1.205621e-07 * 4.329192e-22 * 2.430607e-07 * 1.004298e-01 1.788216e-05 * 9.037688e-10 * |
| **Vector Competence - Legs** | | | |
| **Coefficient** | **Estimate** | **Std. Error** | **P-Value (* <0.05)** |
| (Intercept)  days  speciesAl  temp_scaled  I(temp_scaled^2)  days:speciesAl  days:temp_scaled speciesAl:temp_scaled days:speciesAl:temp_scaled | 0.5629868 0.1046814 -3.2553568 1.1515627 -0.6883879 0.2294182 -0.1144518 1.1182104 -0.1122890 | 0.14414909 0.01304336 0.21580105 0.13272669 0.03773217 0.02003445 0.01127245 0.22265844 0.01935628 | 9.399704e-05 1.009916e-15 * 2.033035e-51 * 4.092297e-18 * 2.306725e-74 * 2.319571e-30 * 3.205707e-24 * 5.111258e-07 * 6.585631e-09 * |
| **Vector Competence - Saliva** | | | |
| **Coefficient** | **Estimate** | **Std. Error** | **P-Value (* <0.05)** |
| (Intercept)  days  speciesAl  temp_scaled  I(temp_scaled^2)  days:temp_scaled speciesAl:temp_scaled | -2.6570633 0.1963576 -0.3121241 1.6934573 -0.6190419 -0.1248945 -0.2276101 | 0.20776783 0.01620703 0.09080376 0.29784384 0.04980923 0.02202838 0.10656125 | 1.898208e-37 8.733549e-34 * 5.874406e-04 * 1.302611e-08 * 1.835953e-35 * 1.430392e-08 * 3.268306e-02 * |

**S2 Table. Results from parsimonious generalized linear models.**
