# Supplementary figures and images for: Impact of extrinsic incubation temperature on natural selection during Zika virus infection of Aedes aegypti and Aedes albopictus
Source: PLoS Pathog. 2021 Nov 9;17(11):e1009433. doi: 10.1371/journal.ppat.1009433 (PMC8629396; doi:10.1371/journal.ppat.1009433)

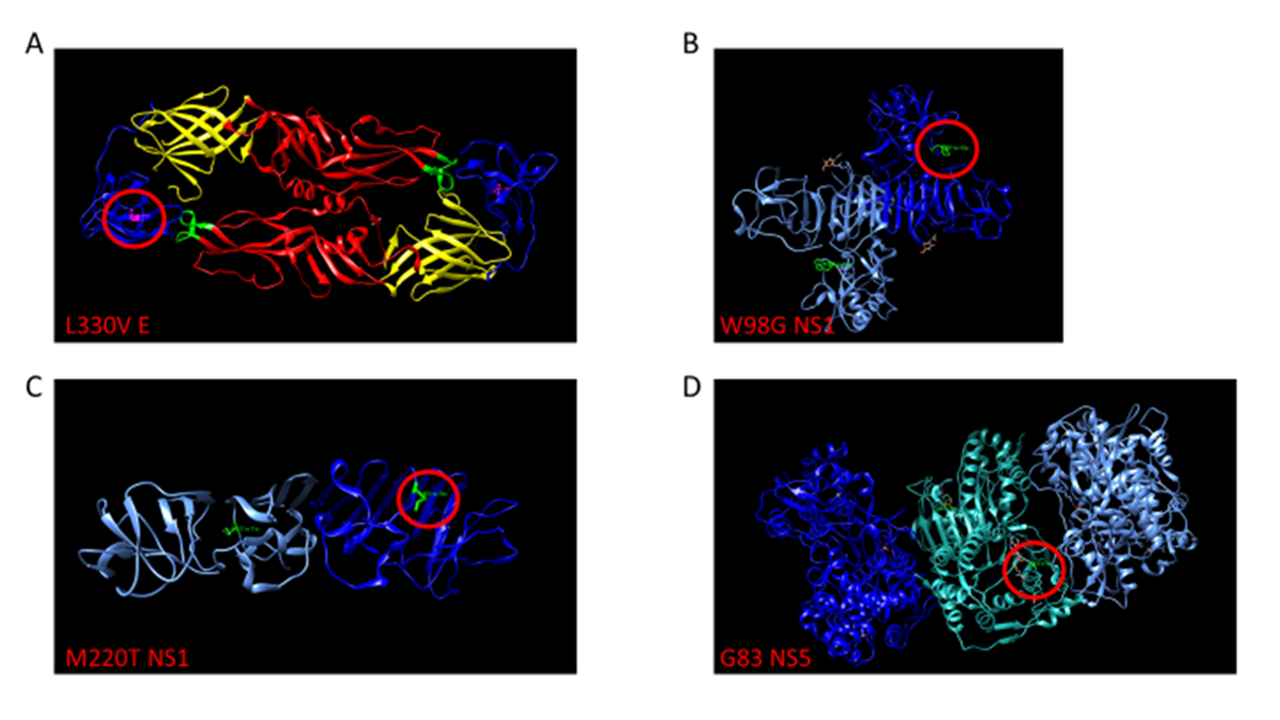

Supplement: S1 Fig — (TIF) [file ppat.1009433.s007.tif]

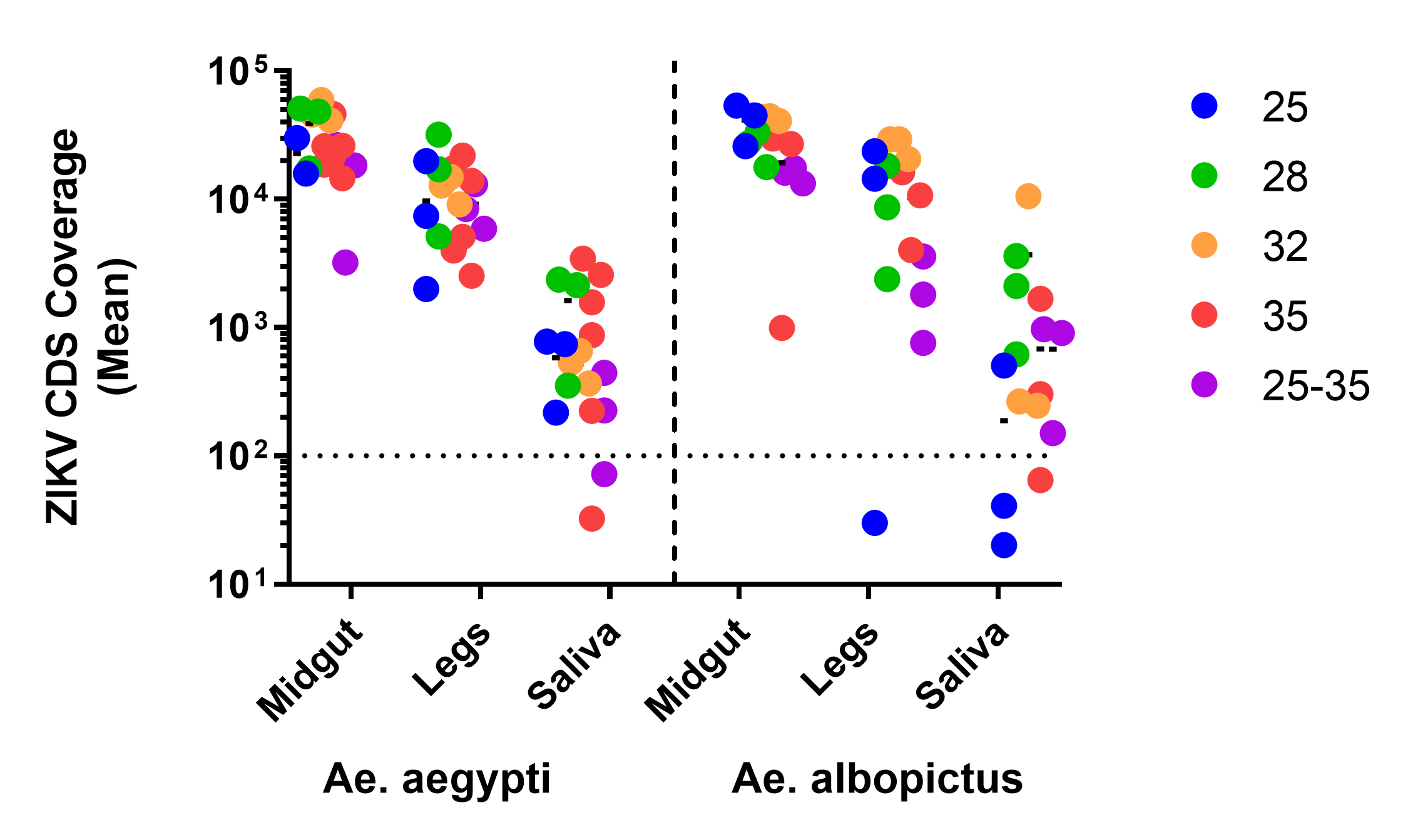

Supplement: S2 Fig — (TIF) [file ppat.1009433.s008.tif]

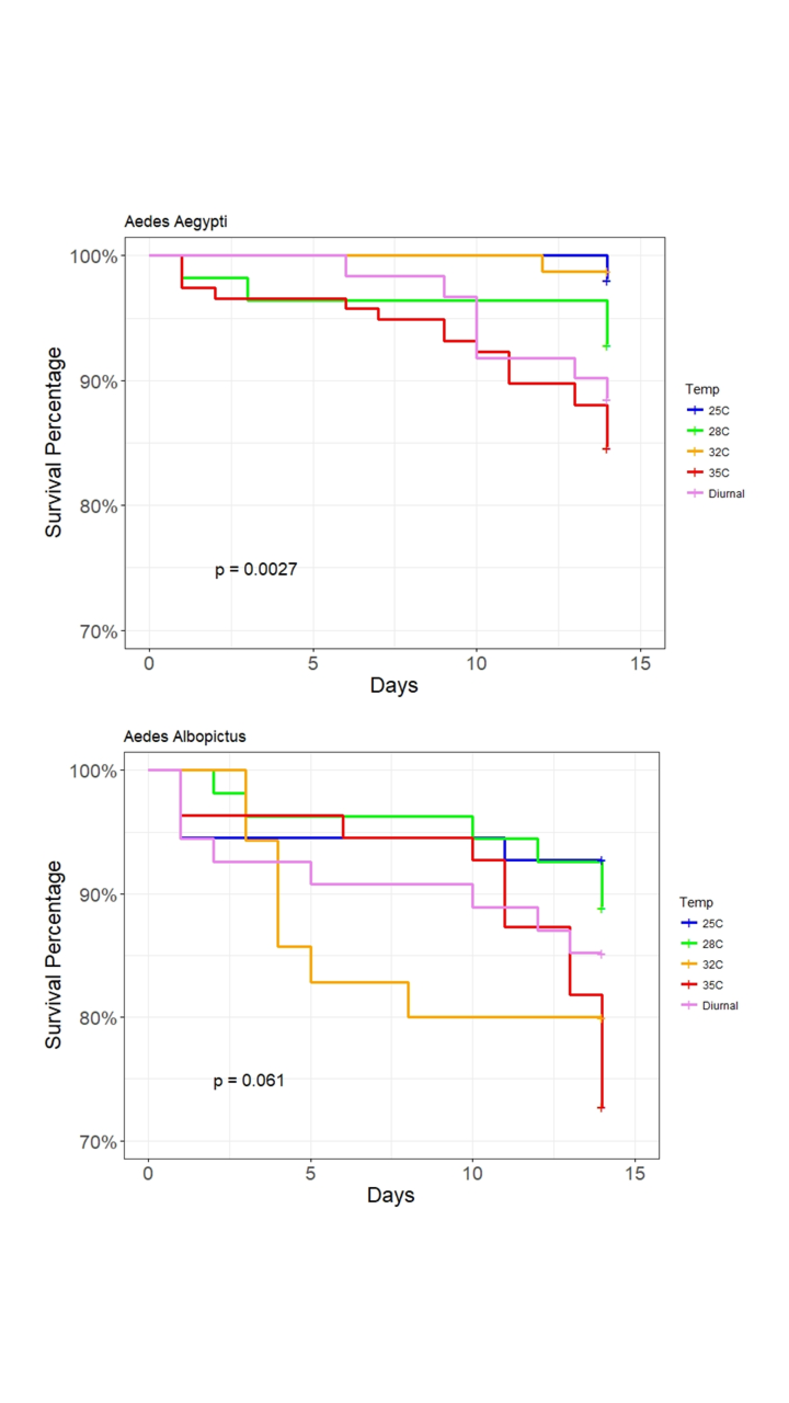

Supplement: S3 Fig — Kaplan-Meier estimate of the survival of ZIKV exposed Ae. albopictus (A) and Ae. aegypti (B) at 5 different temperature groups (25°C, 28°C, 32°C, 35°C and 25°C-35°C). (TIF) [file ppat.1009433.s009.tif]
